# Addressing population heterogeneity and distribution in epidemics models using a cellular automata approach

**DOI:** 10.1186/1756-0500-7-234

**Published:** 2014-04-12

**Authors:** Leonardo López, Germán Burguerner, Leonardo Giovanini

**Affiliations:** 1 Research Center for Signals, Systems and Computational Intelligence, Universidad Nacional del Litoral, Ruta Nacional No 168 - Km 472.4, Santa Fe, Argentina; 2 National Council of Scientific and Technical Research (CONICET), Av. Rivadavia 1917, Buenos Aires, Argentina

## Abstract

**Background:**

The spread of an infectious disease is determined by biological and social factors. Models based on cellular automata are adequate to describe such natural systems consisting of a massive collection of simple interacting objects. They characterize the time evolution of the global system as the emergent behaviour resulting from the interaction of the objects, whose behaviour is defined through a set of simple rules that encode the individual behaviour and the transmission dynamic.

**Methods:**

An epidemic is characterized trough an individual–based–model built upon cellular automata. In the proposed model, each individual of the population is represented by a cell of the automata. This way of modeling an epidemic situation allows to individually define the characteristic of each individual, establish different scenarios and implement control strategies.

**Results:**

A cellular automata model to study the time evolution of a heterogeneous populations through the various stages of disease was proposed, allowing the inclusion of individual heterogeneity, geographical characteristics and social factors that determine the dynamic of the desease. Different assumptions made to built the classical model were evaluated, leading to following results: *i*) for low contact rate (like in quarantine process or low density population areas) the number of infective individuals is lower than other areas where the contact rate is higher, and *ii*) for different initial spacial distributions of infected individuals different epidemic dynamics are obtained due to its influence on the transition rate and the reproductive ratio of disease.

**Conclusions:**

The contact rate and spatial distributions have a central role in the spread of a disease. For low density populations the spread is very low and the number of infected individuals is lower than in highly populated areas. The spacial distribution of the population and the disease focus as well as the geographical characteristic of the area play a central role in the dynamics of the desease.

## Background

The spread of infectious disease is determined by an interplay of biological and social factors [1]. Biological factors are, among others, the virulence of an infectious agent, pre-existing immunity and the pathways of transmission. A major social factor influencing disease spread is the arrangement of potentially contagious contacts between hosts. For instance, the distribution of contacts among the members of a population (*degree distribution*) strongly affects the population spread patterns: Highly connected individuals (a population with a high degree distribution) become infected very early in the course of an epidemic, while those that are nearly isolated (a population with a low degree distribution) become infected very late, if at all [2-4]. If the degree distribution follows a power law, the transmission probability necessary to sustain a disease tends to zero [5-7].

Another important structural property that regulate the spread of diseases is the number of contacts an individual has in a period of time (*clustering of contacts*). High clustering of contacts means higher local spread within cliques and consequently a rapid local depletion of susceptible individuals. In extreme cases, infections get trapped within highly cohesive clusters. Random mixing is known to overestimate the size of an outbreak [8], whereas the local depletion caused by clustering remarkably moderates the rates of disease spread [9,10]. For most of the diseases transmitted by close physical contact, the number of contacts that can be realistically made within the infectious period has a clear upper limit. The mean value of potentially contagious contacts can be interpreted in a meaningful way, since the distribution of daily contacts is unimodal with a “*typical*” number of contacts [11,12]. Recent studies combining survey and modelling showed that the repetition of contacts plays a relevant role in the spread of diseases transmitted via close physical contact [13]. On the other hand, the impact of repetitiveness seems to be negligible in case of conversational contacts [14]. However, the generality of these findings is limited, as they are based on a small, unrepresentative sample and as the specific patterns of such contacts vary depending on the national and cultural context [15].

Many of existing epidemic models employ differential equations explicitly or implicitly [12,13], and do not take into account spatial factors such as variable population density and population dynamics. This kind of models incorporates the *homogeneous mixing* assumption, which is equivalent to a model in which all individuals in a population make contact at an identical rate and have identical probabilities of disease transmission. Although this assumption is unrealistic, it facilitates the mathematical analysis and it consistent with several scenarios for the individual–to–individual transmission. Some authors have relaxed this assumption, but not eliminated from their models [13-20].

In the real world, populations are heterogeneous in features such as susceptibility, infectiousness, contact rates or number of partners. Simple homogeneous mixing models do not allow deviations in host parameters. Heterogeneity in susceptibility and infectivity are important features of many infectious diseases and have been considered to improve the accuracy of epidemiological models. The focus has been placed on the impact of heterogeneity in the final size of epidemics, its consequences on disease control and data interpretation [13,15,21,22]. It has been shown that the final size of the epidemic is reduced when the risk of infection is heterogeneously distributed. There are models that capture some, but not all, of these features [23-29]. A model of an epidemic should incorporate aspects like: *i*) individuals had contact with only a finite number of other individuals, *ii*) contacts that can result in disease transmission are usually short and repeated events, *iii*) the number and frequency of contacts between individuals is not uniform, *iv*) the numbers and identities of an individual’s contacts will change as time goes by and *v*) the individuals have different potential for transmiting a pathogen than its susceptibility to it.

Cellular automata models can fill these aspects and have been used by several researchers as an alternative method to model and simulate epidemies. A cellular automata is formed by *i*) a *n*–dimensional array of identical objects called cells, which are endowed with a state that changes in discrete steps of time according to specific rules, and *ii*) an updated function determines how cells interact with their neighbours, influencing the global behavior of the system [30-33]. In the current literature there are many implementations of epidemic models based on celullar automaton [13,31,34-37]. The ways of approaching the modeling are diverse and can be grouped into different categories according to the relevant features of the model (continuous or discrete space, time or individuals, among others). There are many works in which each cell is considered as a homogeneous distribution of individuals or represent areas of equal size containing a specific population [37,38]. Different cells have different densities and possibly different mobility properties. Infection occurs through contact between individuals of the same cell or neighboring cells. Differential equations are explicitly included in cells and the temporal evolution of the epidemic in each cell follows the classical model, with the modifications that arise from the passage of individuals from a particular cell to another. However, taking all the automata as a whole, the temporal evolution of the epidemic is similar to the classical model. Many models use deterministic rules for updating the cells, although probabilistic rules seem to reflect a more realistic behaviour.

In order to address these issues, we introduced an individual–based–model built upon cellular automata that include all the features described in previous paragraphs. This model allows us to capture the individual heterogeneity as well as a realistic model of individual contacts, modeling individuals explicitly exposed. Each individual is characterized by its own infectivity, suceptibility, contact rate, duration of the contacts and social networks, which defines an individual intrinsic reproductive number *R*_*i*_*i*=1,2,…,*N*. In this paper firstly we will describe how the proposed model was implemented, and then it is employed to characterize the influenza pandemic of 1918 in Geneva [12]. Finally, using the model developed for the influenza pandemic different mitigation strategies were implemented and analyzed for some possible scenarios.

## Methods

Our model is based on a cellular automata and its parameters were established using the information collected about influenza pandemic in the Swiss canton of Geneva in the early twentieth century and modelled before by other authors [12,39]. The programming environment employed to implement and evaluate the model was MATLAB, because it has several advantages over other programming environments: *i*) built–in mathematical and manipulation operations on multi–dimensional arrays, *ii*) user friendly graphical interactive tools, and *iii*) a set of toolboxes that provide specialized optimization and parallel computation functions. Additional file 1 provides the scripts made in MATLAB of the model, and Additional file 2 provides a help file for a correct use of the scipts (see Additional files section).

The main features of the proposed model are: 

• Each cell represents an individual in one of the possible states, or the state of empty cell. No distinction is made between the state of the deceased and the empty cell. Births involve passing from empty cell to a susceptible state.

• The transition between states is probabilistic.

• The initial spacial distribution is random, provided the assumption of homogeneous distribution for large population sizes and thus validate the classic approach. However, user–defined spatial distributions can be also employed.

• The movement of individuals is modeled through a reciprocal change in state neighbouring cells. At first random motion was employed since it emulates the movement that contributes to a homogeneous spatial distribution and contacts between infectious and susceptible individuals (homogeneous mixing assumption). However, user–defined pattern movements can be incorporated to the model.

• Potentially infectious contact is made between infectious individuals and susceptible within the neighbourhood defined as a *zone of influence*.

• For simplicity, the grid type used is rectangular, with Moore neighbourhood and variable size. However, an user–defined grid can be employed to model more complex situations.

• The boundary condition is fixed, with a contour consisting of non interacting empty cells, compatible with the situation in a city, an area of high population density surrounded by a lower density area.

• The simulation progresses in discrete time t given by *t*=*ndt*, with n∈ℝ.

Each cell is then defined as a stochastic Moore machine $A=\left(X,U,Y,d\right)$[40] where: 

• *X* is the set states that comprises six possible conditions: *S* (susceptible), *E* (exposed), *I* (infectious), *A* (Asymptomatic), *R* (Recovered) and *D* (Dead or empty).

• *U* is the set of input. An automaton receives input only when *X*=*S*, issued by another with *X*=*I* or *X*=*A*. When the automaton is in the vicinity of the issuer. Transitions that do not involve contact with infectious individuals are made in probabilistic form independently of a possible entry (transitions to an empty entry *e*).

• *Y* is the output set of *U* issued in state *I* or *A*, corresponding to the input received in state *S*. The output corresponds to the infection probability from contact that has the automata in stage *I* or *A*, obtained from distributing the *b* value for that automaton in the neighbourhood under consideration.

• *d* is the state transition function, which applied to the active state at iteration *k*, the state decides probabilistically active at iteration *k*+1. The function is applied to each cell in two steps: *i*) the state change and recovery from infection and *ii*) the movement.

• For each element of the matrix the probability of moving from state *i* to *j* in each time step, and placing the states *S*,*E*,*I*,*A* and *D* in increasing order from row or column 1 to 6, is defined the transition matrix for empty entry (see Table 1).

**Table 1:** Transition matrix for empty entry

	***S***	***E***	***I***	***A***	***R***	***D***
*S*	1-*μ*	*β*	0	0	0	*μ*
*E*	0	1-(*ε*+*μ*)	*ερ*	*ε*(1-*ρ*)	0	*μ*
*I*	0	0	0	0	*γ*_1_	*μ*
*A*	0	0	0	0	*γ*_1_	*μ*
*R*	0	0	0	0	1-*μ*	*μ*
*D*	*μ*	0	0	0	0	1-*μ*

The main parameters that define the dynamics of disease in the model become from a classical *SEIR* model and they are: *β* is the infection probability for an individual who is in a susceptible state and comes from the infection rate of the classical model, *μ* represents the probability of death, *ε* is the probability of effective contact, determines the rate of infection of individuals, *ρ* determines the rate of passage of from exposed state to infected state, *γ*_1_ is the chance of recovery of infected individuals, *q*: probability of infection from individuals with no symptoms (Asymptomatic), *η* is the birth rate, *λ* is the value of the output function of the automaton, *Ne*: initial number of latent individuals, and *Ni*: initial number of infected individuals. Defining the size of the neighbourhood as *n* and the input value as 1, the transition between the diferent states of an individul is given by the contact transition matrix given in Table 2.

**Table 2:** Contact transition matrix

	***S***	***E***
*S*	1-(*λ*/*η*)	*λ*/*η*
*E*	0	1

The individuals movement is equally likely from a cell centred in an area of predefined size to any other within in this area. The cells swapped positions, which can be interpreted as changes of state. The output function gives the value of infection rate if the automaton is in state *I* or *A*. The initial state vector (*P*(0)) is composed by the probabilities for each initial state given for the automaton, defined as the total number of cells in the grid and as *P*(0)=[*S*_*i*_/*G*,*E*_*i*_/*G*,*I*_*i*_/*G*,*A*_*i*_/*G*,*R*_*i*_/*G*,*D*_*i*_/*G*].

Now, the cellular automata is defined by $R=G\left(T,C\right)$ where: 

• The topology *T* is square. The neighborhood is kind Moore, and is only seen for cells in stage *I* or *A*. The boundary conditions are fixed, with a outline consisting of empty and no interacting cells.

• The connection *C* is unidirectional from cells in state *I* or *A* to the cells in a state *S* that are in the neighbourhood. It provides an entry for each cell in *S* and it is comprised by the value of cells in state *I* or *A*. This value is used to make the transition to state *E*. The cells in a state *S*, which are included in several neighbourhoods in a given time step *k*, will have many chances of changing the state as the number of neighbourhoods in which they are included.

We can see all the illness process; first, during infection, if the individual is in infectious state *I* checks the availability of susceptible neighbours with whom to contact. If there is indeed one in the neighbourhood and the likelihood of the event of infection occurs is fulfilled, then the neighbour state changes to stationary or latency *E*. For individuals in asymptomatic state (*status*=*A*) the behaviour is exactly the same changing the infection probability (*status*=*I*) (Algorithm 2).

The transition from exposed to infectious behaviour is summarized in Algorithm 3. The transition from one state to another is fixed upon check two probabilities: *i*) the probability of transition to infectious state (*status*=*I*) and *ii*) the probability of transition to asymptomatic state (*status*=*A*). Given that a cell is currently in exposed state (*status*=*E*), the algorithm checks the probability of becoming infected. In case of the probability verifies the infectious state the individual becomes infected (*status*=*I*), otherwise the algorithm checks the probability of becoming asymptomatic. Like in the previous case, if the probability verifies the infectious state the individual becomes asymptomatic (*status*=*A*). An infected individual can be reported (*status*=*J*) or not (*status*=*I*) (Algorithm 4). During the recovery phase, if the individual is in infectious state (*Status*=*I*) or asymptomatic state (*Status*=*A*) and satisfies the likelihood of recovery, the individual passes to recovered state (*Status*=*R*) (Algorithm 5). In death by illness, for infectious individuals that satisfies the probability of death, then goes to dead state (*state*=*D*) or empty.

Death by disease is probabilistic, where this probability was adjusted according to the epidemic data. If an individual meets the probability of death by disease is removed, leaving an empty cell in the grid (Algorithm 5). In the case of natural deaths, if the current cell is not empty and satisfies the probability of natural death, then goes to dead or empty state (*State*=*D*), if the cell is in empty, and holds the probability of birth, then it switches to susceptible state (*Status*=*S*). The odds of births and natural deaths are equal, so this process does not affect in the first instance the size of the population (Algorithm 6).

Finally, in the movement phase two cells exchange their values. Firstly, a perturbation for each individual is computed to determine the direction of the movements. Then, the curent state of each cell (corresponding to each individual) is stored in auxiliary variables, which is transfered to the new cell occupied by the individual. This process is repeated with each individual of the population and an individual can move more than once in the same cycle (Algorithm 7).

### Algorithm 1 **Cellular automata dynamics**

The algorithm that simulates the dynamic of the epidemic is summarized in Algorithm 1, and each step is described in Appendix Appendix A. Algorithms. Some of its advantages are: 

• Allows us to control the degree of heterogeneity of the model by defining each individual as a cell within the grid with its own parameters.

• Allows us to model different spatial distributions, resembling the grid topology to that of a real city.

• Directed movement can be implemented to generate different topologies of connection network, which allows to evaluate how the spatial distribution affects the spatio–temporal evolution of the desease.

• Preventive measures can be easily modelled to assess their effects on the evolution of the disease.

• The relaxation of the assumptions of the classical model allows us to study their impact on the spatio–temporal evolution of the desease.

For the model parameters adjustment (see Table 3) we must consider several issues that arises due to the approach. First, there is the problem of scale to be used, the larger the grid and population employed, the closer we get to the large population assumption of the classical model and the computational cost increases. Therefore, it is non desirable to use grid sizes to big for temporal behaviour analysis of the epidemic, however it is used in this paper for the purpose of validating the model with parameters of the classical model. The size of the neighbourhood, determines the degree of global influence. The larger the neighbourhood size used, the closer the results to the assumption of spatial homogeneity of classical model. This is because a very large neighbourhood can influence the infectious even in low density areas of infectious,*softening* the temporal dynamics. Finally, the infectiveness of each individual was modeled using a normal probability distribution which contributes with the population heterogeneity for infective individuals, resulting in a grid full of different types of spreaders differentiable in two populations, one being more active than the others.

**Table 3:** Model parameters

**Parameters**					
***β***	***ρ***	***γ***_**1**_	***q***	***Ne***	***Ni***
8.32	0.082	0.418	0	365	186

**Figure 1:**
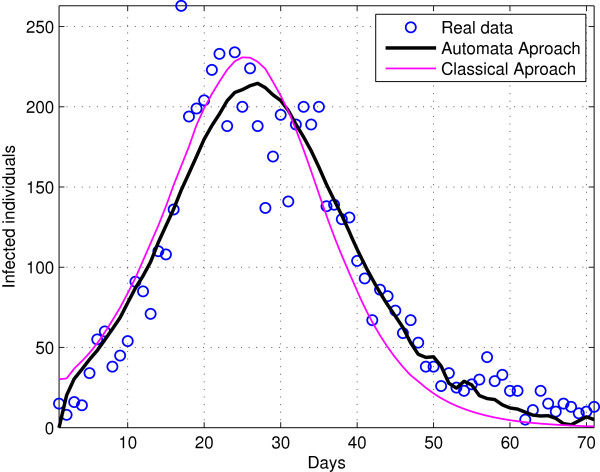
**Epidemic evolution for a large and completely homogeneous population.** In figure we can see in blue real data; black line corresponds to automata model result and red to ODE based model.

To test the capabilities of the proposed model, a comparison with the classical model obtained by Chowell et al. [12] was performed. Firstly, the parameters of the individual–based–model were adjusted by minizing the mean square error between the output predicted by the model and the epidemic data, like in [12]. The model was adjusted to the first wave of the epidemic and the resulting parameters are shown in Table 3. Figure 1 shows the time evolution of infected populations obtained with the proposed model and the classical model [12]. Although, both model have similar mean square error (0.07 for the classical model and 0.068 for the proposed model) the proposed model is able to better approximate the real dynamics of the epidemic because its spatio–temporal modeling capabilities and its multiagent nature.

## Results and discussion

The proposed model allows us to establish and evaluate different scenarios to assess the dynamics of an epidemic and thus determine how each factor influences the population dynamics. To evaluate the model under realistic conditions, simulations were performed with real population size (*N*=75.000) and a neighbourhood around the grid size, using the parameters obtained for the classical model (Table 3). The initial distribution of individuals is kept uniform over the entire domain. The heterogenity of the infection rate was characterized using a normal distribution of suceptibility and infectivity of individuals. Finally, asymptomatic individuals were included in the population, they have a very low infective rate but influence the dynamics of the epidemic as they represent a significant portion of the infected population.

**Figure 2:**
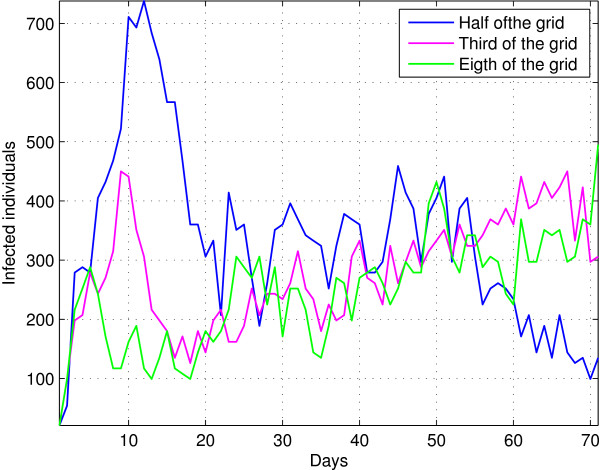
Effect of initial spatial distributions for infected individuals.

Another assumption made in population models is the spatial homogeneity. However, in the case of an epidemic began in one or a few bounded regions, homogeneity could not be achieved instantly if we assume random movement. Varying the initial spatial distribution of infectious individuals, the behaviour of the epidemic break away from the behaviour resulting from classical models (see Figure 2). In the first case we consider infectious individuals initially distributed in one half of the grid.The evolution of the infected population shows a similar behaviour to the classical model but it reaches a lower peak value and a higher stationary number of infected individuals. This phenomenon is caused by the confinement of infectious individual to a smaller area that leads to higher frequency infectious encounters with suceptible individual than expected in homogeneous conditions until the suceptible population is depleted. Then, a high number of suceptible individuals are found when infected individuals move through the grid. In the second case, infectious individuals initially occupy one quarter of the grid. In this case, the evolution of the infected population deviates from the behaviour of the classical model. The number of infected remains at a low but persistent number through the time. This behaviour is the result of the small number of susceptible individuals available in the neighbourhood at all time, which leads to a small reproductive number. However, the infected individuals are able of finding new susceptible as they move through the grid. Finally, infectious individuals only occupy one eighth of the grid. In this case, the evolution of the infected population follows a similar behaviour to the previous case. The results of these simulations are shown in Figure 2. Simulations for the third case but with directed movement (from one corner to the rest of the grid) show a partial recovery of the expected behaviour but delayed several days.

**Figure 3:**
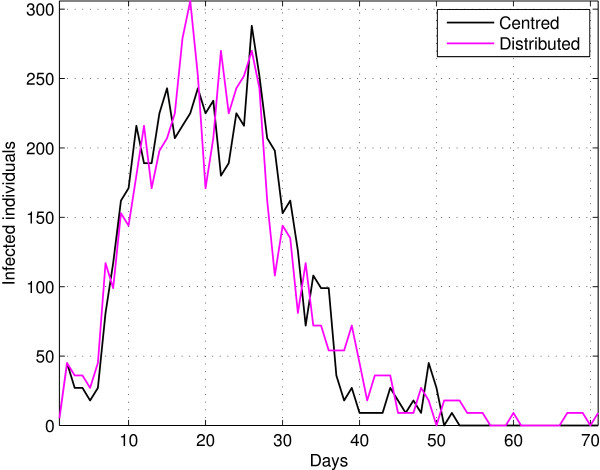
Dynamics for high concentration spatial distribution and uniform concentration spatial distribution and low density.

Now, let us consider a scenario that emulates a real city with a populated urban center surrounded by a peripheral area of lower population density. In this case we analyze the effect of spatial distribution of population through the urban area, considering two spatial distributions: *i*) random concentration and *ii*) uniform concentration (see Figure 3). The evolution of populations for both situations shown similar behaviour, however for the case of homogeneous distribution exhibits a pick in the number of infected individual around day 20 that is greater compared with the other case dynamics but in contrast on the other hand shows a response that is more perdurable in time. In the case of the concentrated population population (i.e urban center) the number of infected people is similar but smaller than the first case, this may be due to the fact that soon infected individuals keep surrounded by recovered individuals stopping the illness process earlier. Both situations shows similar dynamics, in the first case, the number of initial contacts is greater due to the nature of the distribution throwing a longer epidemic.

**Figure 4:**
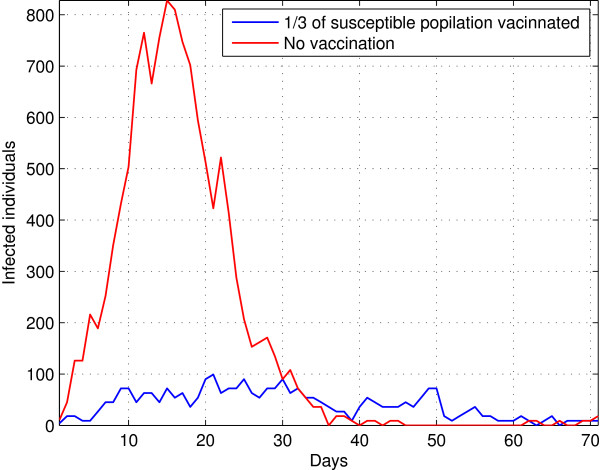
Vaccination effect in disease dynamics for diffrerent vacinnated population size.

A strategy typically used to control an epidemic is vaccination. To assess their effect on the temporal dynamics different populations proposed initial immune individuals. The effect of vaccination plan for an urban population with high density is a drastic decrease in the number of new cases because the initial susceptible population level is considerably lower (Figure 4). In this way, the infected individuals on average have less contact with susceptible, reducing the reproductive number for each generation.

**Figure 5:**
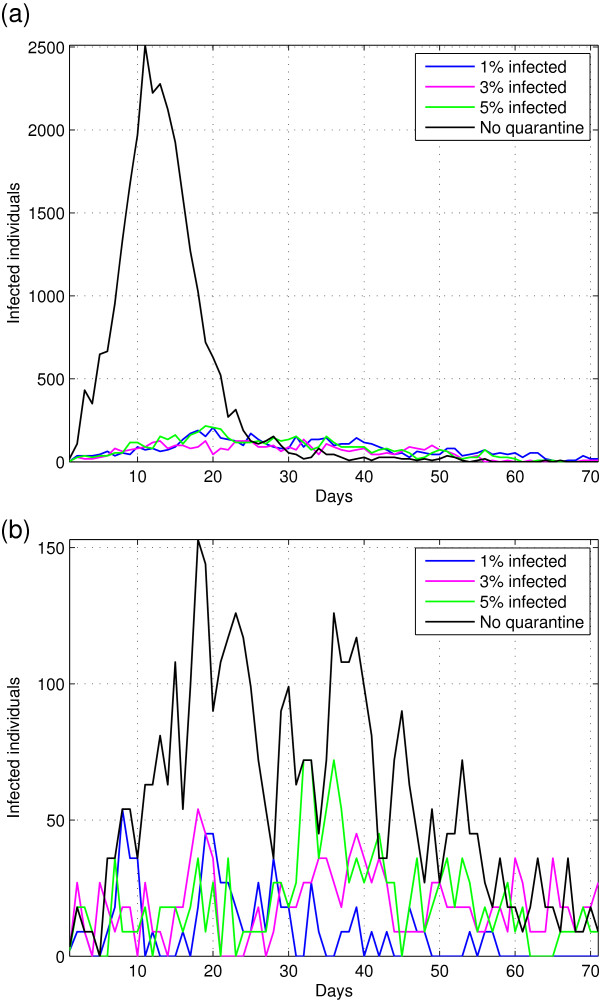
Effect of quarantine on the dynamics of the disease; (a) quarantine at a high population density; (b) quarantine at a low density population.

Finally the last scenario considered in this work is a quarantine.This strategy is usually analysed in other works, is expected to limit the movement of people after a certain period affect disease dynamics. In our model, population stops moving after the number of infected individuals reaches or exceeds a certain threshold (1%, 3%, 5% of population infected). The results of these simulations are shown in Figure 5. Reducing the mobility of individuals seems to work much better when you have highly dense populations like populated urban centres, since in lower dense population the quarantine is implicitly located.

The individual–based–model proposed in this work allows to assimilate into the model features (like individual heterogenity, social behaviour, spatial distribution and geographical features) that improve its accuracy and modeling capabilities. These facts result in more realistic and accurate system representation, as well as a flexibility to define the computational model. The price paid to achieve these advantages is the increment of computational resources required to run the model, compared with the classical model. However, this problem is not significant nowadays due to the reducing cost of computers. It is also important to highlight some drawbacks of the programming enviroment employed to implement the model. MATLAB significantly simplify the implementation mathematical, manipulation and graphical operations required by the model. However, it uses a large amount of computational resources (memory and processor time) that results in large computational times that limit the size of the problem addressed. This drawback can be addressed using a more efficient programming enviroment (like Phyton or C++), but this is not the objective of this work.

## Conclusions

The development of an individual–based–model based on cellular automata provide a powerfull tool to implement computational models that include the relevant features of the epidemic process. The improvement in the modeling capabilities, by including individual characteristics (heterogeneity, spacial distribution and social behaviour) and geographical features, more realistic and accurate dynamic behaviors can be achieved, which translates into better analysis of the system under study.

Finally, it can be noted that the importance of this type of modelling lies in the fact that it allows to analize a complex system (like an epidemic situation) as the interaction of a collection of simpler subsystems, each of one contributes to overall system behaviour. Heterogeneity and spatial structure are relevant properties of such complex systems whose role in the emergent behaviour is necessary to understand. This model is a step forward in this direction since it allows to control all the relevant features identified through this work (individual heterogenity, spatial distribution, social behavior and geographical features) and even more.

## Appendix A. Algorithms

### Infectious state algorithm

#### **Algorithm 2** Infectious state

### Exposed state algorithm

#### **Algorithm 3** Exposed state

### Reported step algorithm

#### **Algorithm 4** Reported step

### Recovery step state algorithm

#### **Algorithm 5** Recovery step

### Dead by illnes algorithm

#### **Algorithm 6** Dead by illness

### Birth and natural deaths algorithm

#### **Algorithm 7** Births and natural deaths

### Individuals movment algorithm

#### **Algorithm 8** Individuals movment

## Competing interests

The authors declare that they have no competing interests.

## Authors’ contributions

All authors contribute to the model conception. LL and GB developed and implemented the epidemic model, adjusted the parameters of the model and carried out the epidemic study. LG oversaw each stage of the work and helped to draft and revise the manuscript. All authors read and approved the final manuscript.

## Supplementary Material

Additional file 1 Supplementary MATLAB scripts (m files) corresponding to the algorithms. Click here for file

Additional file 2 Explains how to use the model, in case you want to replicate experiments. Click here for file

## References

[B1] Koopman JS **Infection transmission science and models** Jpn J Infect Dis 2005 58 6 6 16377860

[B2] Duerr HP Schwehm M De Vlas S Eichner M C **The impact of contact structure on infectious disease control: influenza and antiviral agents** Epidemiol Infect 2007 135 1124 1132 17288643 10.1017/S0950268807007959 PMC2870680

[B3] Morin BR Medina-Rios L Camacho ET Castillo-Chavez C **Static behavioral effects on gonorrhea transmission dynamics in a MSM population** J Theor Biol 2010 267 35 40 10.1016/j.jtbi.2010.07.027 20670632

[B4] Keeling MJ Rohani P Modeling infectious diseases in humans and animals 2011 Princeton, New Jersey: Princeton University Press

[B5] Kiss IZ Green DM Kao RR **The effect of contact heterogeneity and multiple routes of transmission on final epidemic size** Math Biosci 2006 203 124 136 10.1016/j.mbs.2006.03.002 16620875

[B6] Kitsak M Gallos LK Havlin S Liljeros F Muchnik L Stanley HE Makse HA **Identification of influential spreaders in complex networks** Nat Phys 2010 6 11 888 893 10.1038/nphys1746

[B7] Britton T **Stochastic epidemic models: a survey** Math Biosci 2010 225 24 35 10.1016/j.mbs.2010.01.006 20102724

[B8] Zaric GS **Random vs. nonrandom mixing in network epidemic models** Health Care Manag Sci 2002 5 2 147 155 10.1023/A:1014489218178 11993749

[B9] Miller JC **Spread of infectious disease through clustered populations** J R Soc Interface 2009 6 41 1121 1134 10.1098/rsif.2008.0524 19324673 PMC2817154

[B10] House T Keeling MJ **Insights from unifying modern approximations to infections on networks** J R Soc Interface 2011 8 54 67 73 10.1098/rsif.2010.0179 20538755 PMC3024819

[B11] Anderson RM May RM Anderson B Infectious diseases of humans: dynamics and control, Volume 28 1992 Hoboken, USA: Wiley Online Library

[B12] Chowell G Ammon C Hengartner N Hyman J **Transmission dynamics of the great influenza pandemic of 1918 in Geneva, Switzerland Assessing the effects of hypothetical interventions** J Theor Biol 2006 241 2 193 204 10.1016/j.jtbi.2005.11.026 16387331

[B13] Smieszek T Fiebig L Scholz R **Models of epidemics: when contact repetition and clustering should be included** Theor Biol Med Model 2009 6 11 10.1186/1742-4682-6-11 19563624 PMC2709892

[B14] Lloyd A May R **Spatial heterogeneity in epidemic models** J Theor Biol 1996 179 1 11 10.1006/jtbi.1996.0042 8733427

[B15] Rocha LE Blondel VD **Bursts of vertex activation and epidemics in evolving networks** PLoS Comput Biol 2013 9 3 e1002974 10.1371/journal.pcbi.1002974 23555211 PMC3605099

[B16] Finkenstadt B Grenfell B **Empirical determinants of measles metapopulation dynamics in England and Wales** Proc R Soc Lond. Series B: Biol Sci 1998 265 1392 211 10.1098/rspb.1998.0284 PMC1688869 9493407

[B17] Diekmann O Heesterbeek J Mathematical epidemiology of infectious diseases 2000 Hoboken, USA: Wiley Chichester

[B18] Grenfell B Bjornstad O Kappey J **Travelling waves and spatial hierarchies in measles epidemics** Nature 2001 414 6865 716 723 10.1038/414716a 11742391

[B19] den Driessche PV Watmough J **Reproduction numbers and sub-threshold endemic equilibria for compartmental models of disease transmission** Math Biosci 2002 180 29 48 10.1016/S0025-5564(02)00108-6 12387915

[B20] Watts D Muhamad R Medina D Dodds P **Multiscale, resurgent epidemics in a hierarchical metapopulation model** Proc Nat Acad Sci USA 2005 102 32 11157 10.1073/pnas.0501226102 16055564 PMC1183543

[B21] Miller J **Epidemic size and probability in populations with heterogeneous infectivity and susceptibility** Phys Rev E 2007 76 010101 10.1103/PhysRevE.76.010101 17677396

[B22] Rodrigues P Margheri A Rebelo C Gomes M **Heterogeneity in susceptibility to infection can explain high reinfection rates** J Theor Biol 2009 259 2 280 290 10.1016/j.jtbi.2009.03.013 19306886

[B23] Callaway D Newman M Strogatz S Watts D **Network robustness and fragility: percolation on random graphs** Phys Rev Lett 2000 85 25 5468 5471 10.1103/PhysRevLett.85.5468 11136023

[B24] Strogatz S **Exploring complex networks** Nature 2001 410 6825 268 276 10.1038/35065725 11258382

[B25] Newman M **Spread of epidemic disease on networks** Physical Review E 2002 66 016128 10.1103/PhysRevE.66.016128 12241447

[B26] Newman M Watts D Strogatz S **Random graph models of social networks** Proc Nat Acad Sci USA 2002 99 (Suppl 1 2566 11875211 10.1073/pnas.012582999 PMC128577

[B27] Eames K Keeling M **Modeling dynamic and network heterogeneities in the spread of sexually transmitted diseases** Proc Nat Acad Sci 2002 99 20 13330 13335 10.1073/pnas.202244299 12271127 PMC130633

[B28] Meyers L Pourbohloul B Newman M Skowronski D Brunham R **Network theory and SARS: predicting outbreak diversity** J Theor Biol 2005 232 71 81 10.1016/j.jtbi.2004.07.026 15498594 PMC7094100

[B29] Meyers L Newman M Pourbohloul B **Predicting epidemics on directed contact networks** J Theor Biol 2006 240 3 400 418 10.1016/j.jtbi.2005.10.004 16300796

[B30] Ahmed E Agiza H **On modelling epidemics including, latency, incubation and variable susceptibility** Physica A: Stat Theor Phys 1998 253 2 247 352

[B31] Fuentes M Kuperman M **Cellular automata and epidemiological models with spatial dependence** Physica A: Stat Theor Phys 1999 267 3-4 471 486 10.1016/S0378-4371(99)00027-8

[B32] Beauchemin C Samuel J Tuszynski J **A simple cellular automaton model for influenza A viral infections** J Theor Biol 2005 232 2 223 234 10.1016/j.jtbi.2004.08.001 15530492

[B33] White S del Rey A Sánchez G **Modeling epidemics using cellular automata** Appl Math Comput 2007 186 193 202 10.1016/j.amc.2006.06.126 PMC7127728 32287494

[B34] Mansilla R Gutierrez J **Deterministic site exchange cellular automata model for the spread of diseases in human settlements** Arxiv preprint nlin/0004012 2000

[B35] Fu S Milne G **Epidemic modelling using cellular automata** Proc. of the Australian Conference on Artificial Life 2003

[B36] Liu Q Jin Z Liu M **Spatial organization and evolution period of the epidemic model using cellular automata** Phys Rev E 2006 74 3 031110 10.1103/PhysRevE.74.031110 17025597

[B37] Santos L Costa M Pinho S Andrade R Barreto F Teixeira M Barreto M **Periodic forcing in a three-level cellular automata model for a vector-transmitted disease** Phys Rev E 2009 80 016102 10.1103/PhysRevE.80.016102 19658769

[B38] Liu QX Wang RH Jin Z **Persistence, extinction and spatio-temporal synchronization of SIRS cellular automata models** 2008 [http://arxiv.org/abs/0809.1968]

[B39] Chowell G Ammon CE Hengartner NW Hyman JM **Estimating the reproduction number from the initial phase of the Spanish flu pandemic waves in Geneva, Switzerland** Math Biosci Eng 2007 4 3 457 17658935 10.3934/mbe.2007.4.457

[B40] Chopard B Droz M Cellular automata modeling of physical systems 1998 Cambridge, UK: Cambridge University Press

