# Supplementary figures and images for: Addressing population heterogeneity and distribution in epidemics models using a cellular automata approach
Source: BMC Res Notes. 2014 Apr 12;7:234. doi: 10.1186/1756-0500-7-234 (PMC4022236; doi:10.1186/1756-0500-7-234)

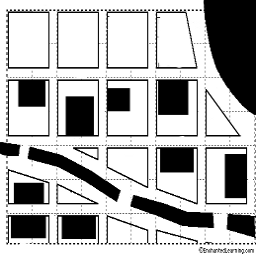

Supplement: Additional file 1 — Supplementary MATLAB scripts (m files) corresponding to the algorithms. [file 1756-0500-7-234-S1.zip › Maps/map.bmp]

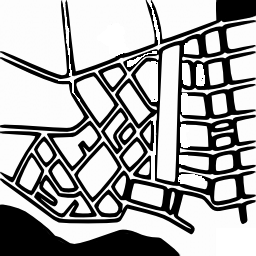

Supplement: Additional file 1 — Supplementary MATLAB scripts (m files) corresponding to the algorithms. [file 1756-0500-7-234-S1.zip › Maps/ciudad.bmp]

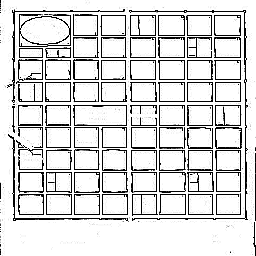

Supplement: Additional file 1 — Supplementary MATLAB scripts (m files) corresponding to the algorithms. [file 1756-0500-7-234-S1.zip › Maps/plano.bmp]

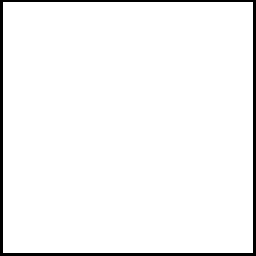

Supplement: Additional file 1 — Supplementary MATLAB scripts (m files) corresponding to the algorithms. [file 1756-0500-7-234-S1.zip › Maps/grill01.bmp]
