# Supplementary material for: Addressing population heterogeneity and distribution in epidemics models using a cellular automata approach
Source: BMC Res Notes. 2014 Apr 12;7:234. doi: 10.1186/1756-0500-7-234 (PMC4022236; doi:10.1186/1756-0500-7-234)
Supplement: Additional file 2 — Explains how to use the model, in case you want to replicate experiments. [file 1756-0500-7-234-S2.pdf]

The next code corresponds to the dynamics of influenza virus, on this script we invocate each function corresponding to each phase of dynamics.

INITIAL PARAMETERS

- **parametros_ini()** function load model parameters, a change in the parameters can change some inputs in the simulation. This is an overload function described later.
- **dist()** returns:
  - pGauss: values of the normal distribution
  - t: time vector associated.
- In **par01** we load:
  - b infection related parameter
  - d fraction of each day.
  - r fraction latent rate become infectious
  - g recovery rate
  - n birth rate and natural death rate
  - d death rate by illness
- In **par02** we load:
  - F: number of rows in the grid
  - C: number of columns in the grid
  - Ns: Number of initial susceptible population
  - Ne: Number of initial latent population
  - Ni: Number of initial infectious population
  - Nr: Number of initial recovered population
- In **par03** we load parameters for visualization:
  - S: Susceptible
  - E: Latent
  - A: Asymptomatic
  - I: Infectious
  - R: Recovered
  - D: Death
  - Vacio: Empty
  - obs: Obstacle

FUNCTION DESCRIPTION:

parametros_ini(escala,vacin): This function sets the initial and general values of the simulator variables, the user can set two of them:

- **parametros_ini**(N) the simulation scale in a range from 1 to N;
- **parametros_ini**(N,1/N) can set a proportion of vaccinated population in a range from 0 to 1/N.

[pGauss t1]=**dist**()

This function calculates the probabilistic distribution for infected individuals, corresponding to a normal distribution.

- **pGauss**: return the values of the probabilistic distribution.
- **t1** return the value of the temporal associated vector.

[grilla grillaI]=**inicializar**(dist,t,grilla,grillaI,sec)

This function set the initial state of the grid.

- Outputs:
  - **grilla**: returns the initial spatial individuals distribution in the grid.
  - **grillaI**: returns the initial map of infective individuals.
- Inputs
  - **dist**: probabilistic distribution of infectious individuals.
  - **t**: time vector associated wit dist.
  - **grilla**: empty grid.
  - **grillaI**: empty density grid.
  - **sec**: the section in the grid where will be distributed the infectious and asymptomatic individuals: 1- all the gird; 2-half of the grid; N-1/N of the grid.

[grilla grillaI]=**dinamica**(grilla,grillaI,dist,x1)

This function describes epidemic dynamics, result are given in the returned grids.

- Outputs:
  - **grilla**: returns the spatial individuals distribution in the grid in the instant t.
  - **grillaI**: returns a map of infective individuals in the instant t.
- Inputs:
  - **grilla**: spatial individuals distribution in the grid in the instant t-1.
  - **grillaI**: a map of infective individuals in the instant t-1.
  - **dist**: probabilistic distribution of infectious individuals.
  - **x1**: time vector associated wit dist.

This function is divided in 5 phases:

- **Infective process.**
- **Latent infective phase.**
- **Infectious phase.**
- **Recovery phase.**
- **Births**
-

[grilla grillaI]=**movimiento**(grilla,grillaI,mov,t,tc)

This function corresponds to the movement phase, the results are given back in the two grids (grilla and grillaI), the parameter mov corresponds to the movement option 0- Random movement and1- directed to the center of the grid. The parameters t and tc model an quarantine state; t is the actual time step and tc is the begin of quarantine

- Outputs:
  - **grilla**: returns the spatial individuals distribution in the grid in the instant t.
  - **grillaI**: returns a map of infective individuals in the instant t.
- Inputs:
  - **grilla**: spatial individuals distribution in the grid in the instant t-1.
  - **grillaI**: a map of infective individuals in the instant t-1.
  - **Mov**: 0 for random movement; 1 center directed movement.
  - **t**: the actual time step.
  - **tc**: the time when quarantine begins.

[St Et Rt At It]=**conteo**(grilla,St,Et,Rt,At,It,t)

This function keeps track of different population size in each time step.

- Output:
    - **St**: total susceptible individuals at time t.
    - **Et**: total latent individuals at time t.
    - **Rt**: total recovered individuals at time t.
    - **At**: total asymptomatic individuals at time t.
    - **It**: total infected individuals at time t.
- Input:
    - **grilla**: population grid at time t.
    - **St**: total susceptible individuals at time t-1.
    - **Et**: total latent individuals at time t-1.
    - **Rt**: total recovered individuals at time t-1.
    - **At**: total asymptomatic individuals at time t-1.
    - **It**: total infected individuals at time t-1.
    - **t**: actual time step.

IMPORTANT NOTES:

The maps ere per-charged, if you want to charge another map you must uncomment the code. For example:

```
grilla=load('Maps/grill01.txt');
% grilla=load('Maps/map.txt');
% grilla=load('Maps/ciudad.txt');
% grilla=load('Maps/plano.txt');
```

in this case , the map grill01 is charged, to charge map:

```
%grilla=load('Maps/grill01.txt');
grilla=load('Maps/map.txt');
% grilla=load('Maps/ciudad.txt');
% grilla=load('Maps/plano.txt');
```

For try different population densities

```
parametros_ini(D)
```

so, to set a density of 1/3 of the original size the you must set:

```
parametros_ini(3)
```

and for 1/10 of the original one you must set:

```
parametros_ini(10)
```

For try different initial distributions in the grid for infective individuals then you must set the function inicializar(' '), so, for total grid initial distribution :

```
inicializar(pGauss,t,grilla,grillaI,1);
```

for a half of the grid initial distribution

```
inicializar(pGauss,t,grilla,grillaI,2);
```

for fourth of the grid distribution:

```
inicializar(pGauss,t,grilla,grillaI,4);
```

and so.

For quarantine you must set the las two parameters of the movimiento function, to stop movement in day 10 then:

```
[grilla grillaI]=movimiento(grilla,grillaI,mov,t,10).
```
